# The DNA-Damage Response to γ-Radiation Is Affected by miR-27a in A549 Cells

**DOI:** 10.3390/ijms140917881

**Published:** 2013-09-02

**Authors:** Andrea Di Francesco, Cristiano De Pittà, Francesca Moret, Vito Barbieri, Lucia Celotti, Maddalena Mognato

**Affiliations:** 1 Department of Biology, University of Padova, via U. Bassi 58/B, Padova 35131, Italy; E-Mails: difrancesco_andrea@hotmail.it (A.D.F.); cristiano.depitta@unipd.it (C.D.P.); francesca.moret@unipd.it (F.M.); 2 Department of Surgery, Oncology and Gastroenterology, University of Padova via Gattamelata 64, Padova 35128, Italy; E-Mail: vito.barbieri@unipd.it; 3 INFN-Laboratori Nazionali di Legnaro, Viale dell’Università 2, Legnaro 35020, Padova, Italy

**Keywords:** γ-radiation, DNA Damage Response, miR-27a, *ATM*, A549 cells

## Abstract

Perturbations during the cell DNA-Damage Response (DDR) can originate from alteration in the functionality of the microRNA-mediated gene regulation, being microRNAs (miRNAs), small non-coding RNAs that act as post-transcriptional regulators of gene expression. The oncogenic miR-27a is over-expressed in several tumors and, in the present study, we investigated its interaction with *ATM*, the gene coding for the main kinase of DDR pathway. Experimental validation to confirm miR-27a as a direct regulator of *ATM* was performed by site-direct mutagenesis of the luciferase reporter vector containing the 3′UTR of *ATM* gene, and by miRNA oligonucleotide mimics. We then explored the functional miR-27a/*ATM* interaction under biological conditions, *i.e.*, during the response of A549 cells to ionizing radiation (IR) exposure. To evaluate if miR-27a over-expression affects IR-induced DDR activation in A549 cells we determined cell survival, cell cycle progression and DNA double-strand break (DSB) repair. Our results show that up-regulation of miR-27a promotes cell proliferation of non-irradiated and irradiated cells. Moreover, increased expression of endogenous mature miR-27a in A549 cells affects DBS rejoining kinetics early after irradiation.

## 1. Introduction

To maintain the integrity of genome, eukaryotic cells rely on a highly regulated system pathway of response to DNA damage—the DNA-Damage Response (DDR)—which encompasses damage sensors, mediators, signal transducers and effectors. Upon recognition of DNA damage, transducer kinases ATM (Ataxia-Telangiectasia Mutated), ATR (Ataxia telangiectasia and Rad3 related), and DNA-PKcs (DNA-dependent protein kinase catalytic subunit) relay and amplify the damage signal to effector proteins that in turn activate cell cycle checkpoints, regulate transcription, translation, and metabolism, and activate the appropriate DNA repair process, as well as cell fate toward apoptosis or senescence [1,2]. Following DNA double-strand break (DSB) induction, ATM undergoes spatial relocalization and catalytic activation; after that, it is rapidly recruited to DNA damage sites. Here ATM phosphorylates specific serines or threonines on many downstream protein substrates, including “Ser-139” of histone variant H2AXpresent in nucleosomes surrounding DSB sites, thereby regulating DDR mechanism.

Following genotoxic stress, DDR pathway is post-transcriptionally regulated through selective mRNA stabilization or decay and regulation of translation [3]. In this context, non-coding RNAs, such as microRNAs (miRNAs), have emerged as important regulators of gene expression of key components of DDR pathway. miRNAs are natural single-stranded, small RNA molecules (18–22 nt) that regulate gene expression by binding to target mRNAs and suppress their translation or promote their cleavage [4]. Mature miRNAs recognize their target mRNAs by base-pairing interactions between nucleotides 2–8 of the miRNA (the seed region) and complementary nucleotides in the 3′-untranslated region (3′UTR) of mRNAs. Recent evidences, however, suggest that in addition to 3′UTRs miRNAs can bind to other regions of target mRNAs, including the 5′UTRs, promoter, and open reading frames [5]. In humans, ~800 miRNAs are predicted to exist [6], and each single miRNA can influence the expression of up 1000 genes. miRNAs regulate many physiological processes, including differentiation, apoptosis, fat metabolism, as well as pathological processes, such as tumorigenesis. Different miRNAs are indeed dysregulated in human cancers and can function as either tumor suppressors or oncogenes, by targeting different steps of the tumorigenesis process, including initiation and progression to a metastatic phenotype [7–10]. Moreover, accumulating evidences have shown that miRNAs are altered following genotoxic and cytotoxic stress, and several studies suggested that miRNA expression is regulated in DDR at the transcriptional level, in a p53-dependent manner [11] and through modulation of miRNAs’ processing and maturation steps [12]. Moreover, more than half of the DNA repair and DNA damage checkpoint genes contained conserved miRNA target sites [13].

By integrating the transcriptome and microRNome, we recently identified some miRNA-related genes of DDR pathway that were altered in human peripheral blood lymphocytes irradiated with γ-rays and incubated in ground gravity (1 *g*) and in modeled microgravity (MMG) [14]. Several miRNAs were specifically dysregulated by IR in a dose-, time- and gravity-related manner. Among miRNAs altered by the combined action of radiation and microgravity, we identified miR-27a, which as a result, was anti-correlated to *ATM*. miR-27a is classified as an *oncomir*, being over-expressed in several malignancies, including breast cancer [15], gastric and renal carcinoma [16,17], hepatocellular carcinoma [18] and pediatric B-ALL [19]. Down-regulation of miR-27a has been reported in colorectal cancer [20], in oral squamous carcinoma [21], and in the serum and plasma of non-small cell lung cancer patients [22]. miR-27a is part of a cluster of three miRNAs expressed from an intergenic region of chromosome 19, whose members—miR-23a, miR-27a, miR-24-2—are involved in cell cycle control and differentiation in various cell types [23]. Recent evidences report that miR-23a~24-2~27a cluster may possess a causal role in mammary tumorigenesis, since the expression levels of its members were significantly higher in breast cancer with lymphnode metastasis compared with that from patients without lymphnode metastasis or normal tissue [24]. The increased expression of miR-23a~24-2~27a cluster possesses also important function in neovascular age-related macular degeneration and tumor-related angiogenesis [25], however, the mechanisms of regulation in cancer progression is still poorly understood.

In the current study, we investigated the role of miR-27a in the DDR induced by γ-radiation in lung cancer A549 cells. We first validated the functional interaction between miR-27a and *ATM* by performing site-directed mutagenesis of 3′-untranslated region (UTR) of *ATM* gene. We then analyzed the biological effects of miR-27a over-expression on the DNA damage response to γ-rays in A549 cells by using miRNA mimics which are chemically synthesized double stranded RNAs that, when introduced into cells, efficiently mimic specific endogenous miRNAs.

## 2. Results and Discussion

### 2.1. miR-27a Interacts with 3′UTR of ATM Gene

**Figure 1:**
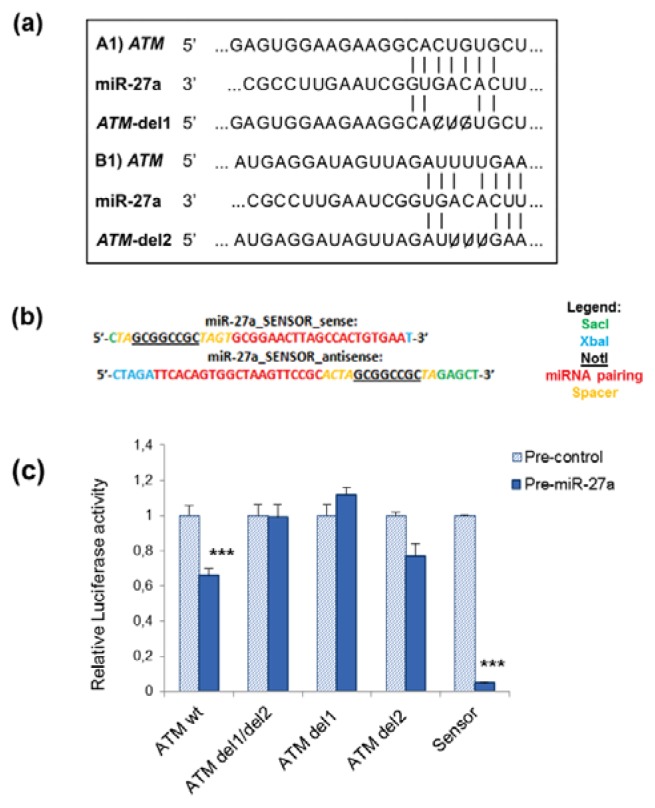
Ataxia-Telangiectasia Mutated (*ATM*) is a target of miR-27a in A549 cells. (**a**) Diagram illustrating the two putative regions of *ATM*-3′UTR for miR-27a binding and corresponding mutant miR-27a binding sites. Two deletions were generated in the *ATM*-3′UTR sequence in the complementary site for the seed regions of miR-27a as indicated; (**b**) Reporter constructs containing a synthetic sequence including three perfect miR-27a binding sites (miR-27a sensor) were generated as described in the Experimental Section; (**c**) Effects of miR-27a binding sites on luciferase activity. A459 cells were co-transfected with the firefly luciferase reporter plasmid containing wild-type or mutant *ATM*-3′UTR, with pre-miRNA precursor (pre-miR-27a) or pre-miRNA precursor-Negative Control (pre-control). Luciferase activity was assayed 24 h after transfection. The data represent mean ± S.D. from three independent experiments, normalized on Renilla Luciferase activity (***p < 0.001, *t*-test); del = deletion; 1 and 2 binding sites. Data of relative luciferase activity of *ATM* wt and miR-27a sensor are from Girardi *et al.* [14].

ATM-mediated DNA damage response is an important barrier to prevent tumorigenesis, indeed ATM down-regulation has been observed in many cancers [26]. We recently demonstrated that miR-27a and *ATM* are differentially expressed and anti-correlated in γ-irradiated human lymphocytes incubated in microgravity and that miR-27a could interact with *ATM*-3′UTR [14]. However, to validate that miR-27a actually targets *ATM*-UTR, in the present study we created luciferase constructs containing wild type and mutated 3′UTR of *ATM* gene, and tested them for miR-27a-*ATM* interaction. The *ATM* gene has a long (~3.7 kb) and highly conserved 3′UTR, and two different putative miR-27a target sites have been predicted by PITA algorithm. We generated two mutations in the *ATM*-3′UTR sequence in the complementary site for the seed region of miR-27a as indicated in Figure 1a. We co-transfected A549 cells with miR-27a precursor (pre-miR-27a) together with the wild-type *ATM*-3′UTR luciferase reporter or with reporter in which the *ATM*-3′UTR sequence was mutated at each individual miR-27a binding site (*ATM*-del1 and *ATM*-del2). In addition, we generated a construct containing the synthetic sequence including the perfect miR-27a binding site (sensor) to be used as positive control for each experiment (Figure 1b). As shown in Figure 1c, the activity of the reporter construct containing wild type *ATM*-3′UTR was significantly decreased by the treatment with miR-27a mimic, while the construct with two seed regions of miR-27a mutated at site 1 was refractory to miR-27a-mediated repression. By analyzing the effect of the two different mutated seed regions on luciferase activity we observed that the miR-27a binding mutated site 1 (*ATM*-del1) showed a real interaction with miR-27a. On the contrary, the miR-27a binding mutated site 2 (*ATM*-del2) showed a decreased luciferase activity, indicating a weak miRNA-mRNA interaction in that position of *ATM*-3′UTR sequence.

### 2.2. miR-27a Affects ATM Expression Levels in A549 Cells

**Figure 2:**
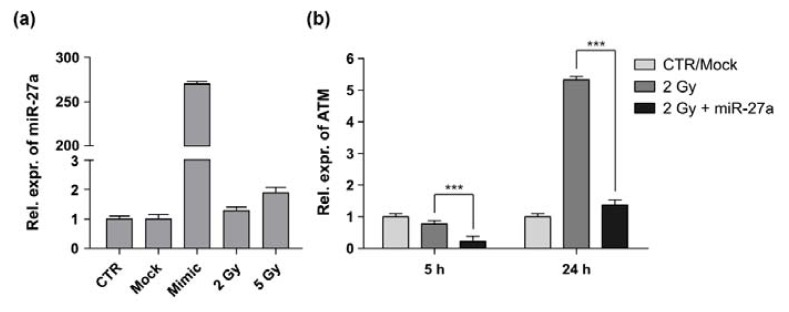
Expression of miR-27a and *ATM* in A549 cells. (**a**) Relative expression of miR-27a measured by qRT-PCR in untransfected cells (CTR), in mock-transfected cells, in miR-27a transfected cells (mimic) and in untransfected cells irradiated with γ-rays (2 and 5 Gy). Analyses have been performed at 24 h after transfection or irradiation; (**b**) Relative expression of *ATM* transcript measured by qRT-PCR in cells transfected or not with miR-27a and irradiated with 2 Gy. Analysis was performed at 5 h and 24 h after irradiation andRNU48 was used as an internal loading control in all reactions. At both time points, the expression level of *ATM* is significantly down-regulated in cells over-expressing miR-27a (***p < 0.001, *t*-test).

The main mechanism of miRNA action is thought to be degradation of mRNA or inhibition of translation [4]; therefore, the effect of a miRNA mimic can be assayed at the mRNA level of its target gene. Before evaluating the action of miR-27a on modulation of *ATM* transcript, we first verified that the miRNA was not endogenously over-expressed in A549 cells and it was properly processed following transient transfection with plasmid and miRNA mimic. The results of quantitative RT-PCR (qRT-PCR) showed that under physiological conditions the mature miR-27a was not up-regulated in A549 cells, whereas it was highly induced after transfection (Figure 2a). At the same time, we verified if IR induced dysregulation of miR-27a by examining its expression level in γ-irradiated cells. The results show that the expression level of miR-27a is almost unaffected in 2 Gy-treated cells and only slightly increased in 5 Gy-treated cells.

The functional interaction between miR-27a and *ATM* transcript was validated by measuring the expression level of *ATM* in non-irradiated and 2 Gy-irradiated A549 cells transfected with miR-27a mimic (Figure 2b). Cells were harvested at 5 h and 24 h after irradiation and the expression level of *ATM* was compared with that of non-irradiated control cells. At 5 h after irradiation, *ATM* expression was slightly decreased in irradiated cells in respect to control cells, in accordance with data of Ghosh *et al.* [27] obtained in A549 cells irradiated with the same dose (2 Gy) of γ-rays. At 24 h after irradiation, *ATM* expression significantly increased (~5-fold) in comparison with control cells. At both time points after irradiation, notably, the level of *ATM* was significantly decreased in cells over-expressing miR-27a. Besides the action of miR-27a on *ATM*-3′UTR, such mRNA reduction could be due to an *ATM* autoregulatory feedback mechanism in response to DNA damage, as recently proposed by Clyde *et al.* [28]. According to these authors, cells have evolved a sensor mechanism that results in rapid induction of the *ATM* transcript to compensate for ATM chemical inhibition through a negative feedback. In contrast, in our experiments, the decreased expression of *ATM* transcript could be related to a positive feedback mechanism that down-regulates *ATM* transcription.

### 2.3. Effects of miR-27a on Proliferation of A549 Cells

**Figure 3:**
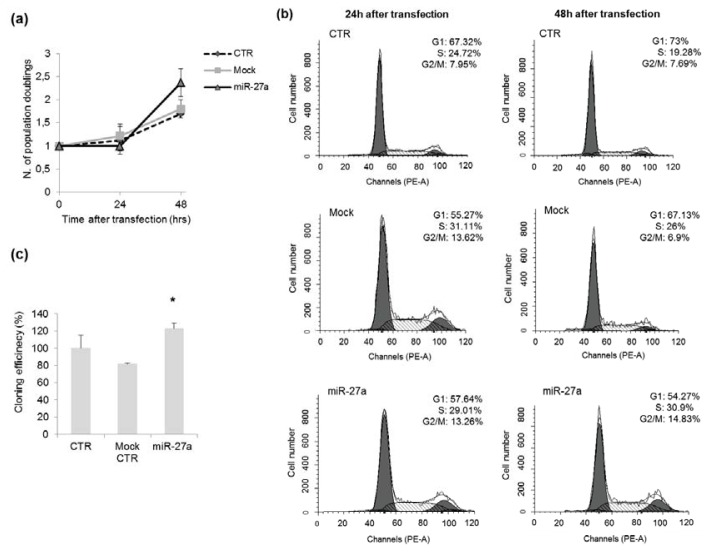
Effects of miR-27a up-regulation on proliferation of A549 cells. (**a**) Number of population doublings in untransfected cells (CTR), cells transfected with miR-27a mimic and mock-transfected cells; (**b**) Flow cytometric analysis of CTR, mock, and miR-27a transfected cells at 24 h and 48 h after transfection; (**c**) Cloning efficiency determined 24 h after transfection in cells over-expressing miR-27a, in mock-transfected and untransfected control cells (CTR, *p < 0.05, *t*-test).

We evaluated the effects of miR-27a over-expression on DDR induced by γ-rays in A549 cells. In cells enforced to over-express miR-27a, the proliferation rate increased, in particular at 48 h after transfection, as indicated by the higher number of population doublings (2.4 in miR-27a transfected cells *vs.* ~1.7 in control cells, Figure 3a). Analysis of cell cycle distribution evidenced perturbations in cell cycle progression of A549 over-expressing miR-27a, as indicated by the increase of cells in S-phase at 48 h after transfection (31% *vs.* 19%–26% in control cells, Figure 3b). This finding is in agreement with the results of Lerner *et al.* [19], showing that miR-27a over-expression significantly increased the number of cells in S-phase in tumor-derived cell lines different from A459. At the same time, we observed an increase of G_2_ cells from ~7% in control cells to ~15% in cells over-expressing miR-27a. We then evaluated if miR-27a up-regulation could influence the colony forming ability of A549 cells. Our experiments show that cloning efficiency was higher in cells over-expressing miR-27a than in control cells (Figure 3c, *p* < 0.05), indicating that the endogenous level of miR-27a can also affect the cloning efficiency of A549 cells.

Our findings are, on the whole, consistent with the activity of miR-23a~24-2~27a cluster in promoting cell proliferation as reported for other cancer cells [29,30]. Over-expression of miR-27a has been observed, indeed, to enhance cell proliferation, promote migration and invasion, and activated cell cycling in hepatocellular carcinoma cells [18]. In gastric adenocarcinoma, cell line miR-27a functions as an oncogene and its knocking down could suppress cell growth [17]. Furthermore, miR-27a has been found to promote cell proliferation also in non-cancer cells such as myoblasts through targeting myostatin [31].

### 2.4. Effects of miR-27a Up-Regulation on Radiosensitivity of A549 Cells

**Figure 4:**
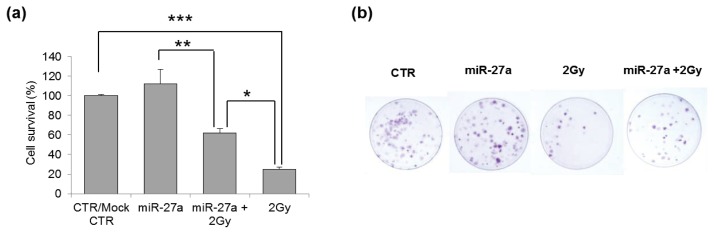
Effects of miR-27a up-regulation on radiosensitivity of A549 cells. (**a**) Cell survival measured by cloning efficiency in control cells (both untransfected cells, CTR, and mock-transfected), in cells subjected to only irradiation (2 Gy), to only transfection with miRNA mimic (miR-27a), and to both transfection with miR-27a mimic and irradiation (miR-27a + 2 Gy); (**b**) Representative image of clones originated from A549 cells. Error bars represent the mean ± S.D. (*p < 0.05; **p < 0.01; ***p < 0.001; *t*-test).

In response to DNA DSBs, the serine-threonine protein kinase ATM coordinates many cellular processes, starting with the phosphorylation of numerous substrates active in various branches of the DDR [32,33]. The phosphorylation of H2AX (γ-H2AX) is one of the earliest ATM-dependent responses to IR followed by the formation of nuclear foci with a critical role in the retention of repair factors at the sites of DSBs [34,35]. To examine the effects of miR-27a on ATM signaling in response to IR in A549 cells, we performed the clonogenic survival assay. Our results show that cells over-expressing miR-27a were less sensitive to IR than their counterparts. As shown in Figure 4a, the up-regulation of miR-27a increased cell survival from 25% to 62% (*p* < 0.05). Our data are in discordance with those reported from breast cancer cells, in which a clonogenic assay showed that ATM down-regulation driven by miR-18a renders cells hypersensitive to IR [36].

**Figure 5:**
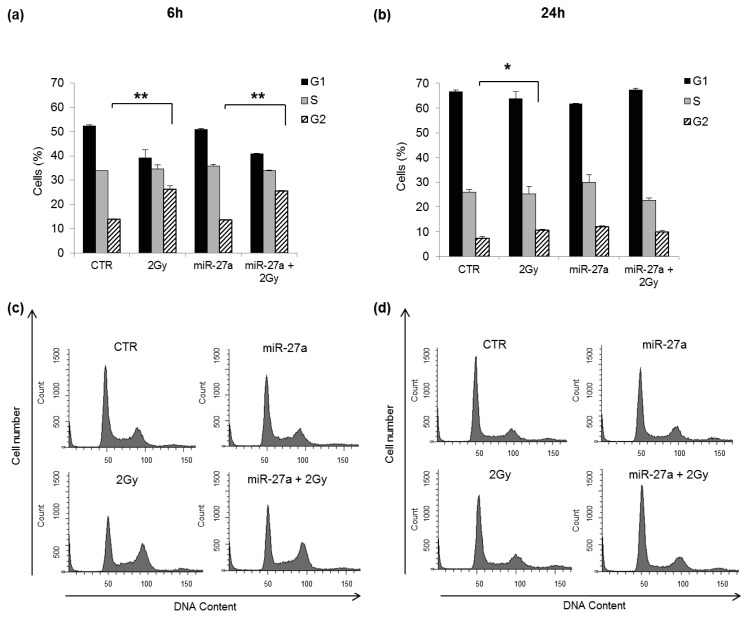
Flow cytometry analysis of cell cycle distribution of A549 cells at 6 h (**a**,**c**) and 24 h (**b**,**d**) after γ-radiation. (CTR, untransfected non-irradiated cells; 2 Gy, untransfected irradiated cells; miR-27a, cells transfected with miR-27a; miR-27a + 2 Gy, cells transfected with miR-27a and irradiated 24 h later). (**a**) Fraction of cells in G_1_-, S- and G_2_-phase at 6 h after irradiation and in non-irradiated control cells. The fraction of G_2_ cells was significantly higher both in miR-27a transfected and untransfected cells, respect to their proper non-irradiated control cells (26% *vs.* 13%, respectively, **p < 0.01, *t*-test); (**b**) Fraction of cells in G_1_-, S- and G_2_-phase at 24 h after irradiation in comparison with their proper non-irradiated control cells. A little G_2_ arrest was present in untransfected irradiated cells (10.7% in 2 Gy cells *vs.* 7.4% in CTR cells, *p < 0.05, *t*-test). Representative histogram plots of cell cycle distribution at 6 h (**c**) and at 24 h (**d**) after IR are shown. Mock-transfected cells have a cell cycle distribution similar to that of CTR cells at both time points (not shown).

Our results could be consistent with the role of ATM in the control of cell cycle checkpoints, in accordance with data from AT cell lines which showed impaired G_1_–S, S, and G_2_–M checkpoint arrest following IR exposure [37,38]. Under normal conditions following the induction of DSBs, ATM activates and stabilizes p53, which in turn drives the expression of genes involved in cell proliferation and apoptosis [39,40]. The higher colony forming ability of irradiated A549 cells over-expressing miR-27a respect to untransfected irradiated cells could be probably related to alterations in the p53-modulation of genes involved in the activation of cell cycle checkpoints, such as p21. Previous studies have shown, moreover, that heterogeneous nuclear ribonucleoprotein K (hnRNPK) is stabilized in an ATM-dependent manner in response to DNA damage and acts as a cofactor for p53-mediated transcription [41,42]. We thus suggest that when *ATM* is down-regulated both p53 and hnRNPK activity are affected, inhibiting or altering the p21-mediated cell cycle arrest. To test this hypothesis, we analyzed cell cycle progression by flow cytometry in A549 cells, over-expressing or not miR-27a, after irradiation with 2 Gy of γ-rays. The results at 6 h after irradiation show the induction of G_2_-phase arrest in untransfected cells and in cells over-expressing miR-27a, without differences between the two cells populations (Figure 5a). At 24 h after irradiation, a little G_2_ arrest was still present in untransfected cells but not in cells over-expressing the miRNA, without significant differences between the two cells populations (Figure 5b). Flow cytometric analyses carried out on the same samples did not detect a sub-G_1_ peak (Figure 5b,d), suggesting that after irradiation with 2 Gy, A549 cells undergo cell cycle arrest rather than apoptosis. According to our findings, the p21-mediated G_2_/M arrest seems unaffected in irradiated A549 cells over-expressing miR-27a, suggesting that the higher colony forming ability of such cells respect to untransfected irradiated cells could be related to a p53-independent mechanism. Indeed, as recently reported by Shin *et al.* [43], p21 can be activated by both p53-dependent and p53-independent mechanisms and can assume cell cycle arresting functions in response to anti-cancer drugs treatment, depending on the cellular context.

**Figure 6:**
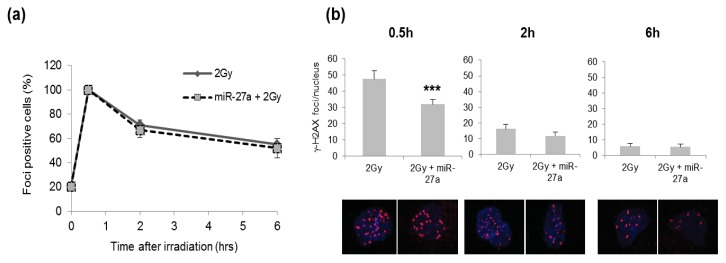
Kinetics of DSB rejoining determined by the percentage of γ-H2AX foci positive cells, over-expressing or not miR-27a, irradiated with 2 Gy. (**a**) Fraction of A549 cells positive for γ-H2AX foci at 0.5 h, 2 h and 6 h after irradiation with 2 Gy; (**b**) Mean number of γ-H2AX foci in 2 Gy-irradiated cells over-expressing or not miR-27a, together with representative pictures of foci in nuclei stained with DAPI at 0.5 h, 2 h and 6 h after γ-irradiation; quantification of foci/nucleus is from at least 200 cells/time point. Error bars represent the mean ± S.D. (***p < 0.001, *t*-test).

It has been reported that IR could up-regulate ATM expression and consequently lead to phosphorylation of H2AX [44]. Since we observed that in irradiated A549 cells, *ATM* was ~5-fold induced in untransfected A549 cells and ~1.3-fold induced in cells transfected with miR-27a mimic (Figure 2b). We analyzed formation and rejoining of DSBs by determining the number of IR-induced foci of γ-H2AX histone in cells that were or were not over-expressing miR-27a. We analyzed the kinetics of γ-H2AX foci in A549 cells that were or were not over-expressing miR-27a, at 0.5 h, 2 h and 6 h after irradiation with 2 Gy of γ-rays, by determining the number of foci positive cells. The kinetics of DSB rejoining appeared very similar in both miR-27a-transfected cells and mock control cells, with a peak of foci induction at 30 min after irradiation followed by a decrease within 6 h from irradiation (Figure 6a). At 30 min after irradiation, however, we found that the mean number of γ-H2AX foci per nucleus was significantly lower in cells over-expressing miR-27a (*p* < 0.01), whereas at later time points, no differences were detected between transfected and untransfected cells (Figure 6b). Our results are in accordance with the reduced γ-H2AX foci formation in response to radiation observed at early post-irradiation times in ATM deficient cells compared with cells expressing wild-type ATM [44,45].

## 3. Experimental Section

### 3.1. Cell Culture

The human A549 cells (lung adenocarcinoma) were purchased from American Type Culture Collection (ATCC n. CCL-185™) and cultured in Ham’s F12-K Nutrient Mixture (Invitrogen Life Technologies, Carlsbad, CA, USA) supplemented with 10% heat inactivated fetal bovine serum (FBS, BIOCHROM, Berlin, Germany), 38 units/mL streptomycin, and 100 units/mL penicillin G, in T75 cm^2^ flasks (FALCON). Cells were kept at 37 °C in a humidified atmosphere of 95% air and 5% CO_2_, and maintained in exponential and asynchronous phase of growth by repeated trypsinization and reseeding prior to reaching subconfluency.

### 3.2. Construction of Recombinant Vectors and Site-Directed Mutagenesis

Luciferase reporter vectors were generated from human cDNA and cloned into the pmirGLO Dual-Luciferase miRNA Target Expression Vector (Promega, Madison, WI, USA), immediately downstream from the stop codon of the luciferase gene. To predict base-pairing, we used PITA algorithm [46], which predicts the targets of a miRNA by searching for the presence of 6-mer or 7-mer sites that near-perfect match the seed region of the miRNA, allowing for G:U wobbles and considering the role of site accessibility in microRNA target recognition. When indicated, the *ATM*-3′UTR was mutagenized at the miR-27a recognition sites using the Quick Change Site-Directed Mutagenesis kit (Stratagene, Agilent Technologies, Santa Clara, CA, USA) according to manufacturer’s instructions. miR-27a sensor was obtained by annealing, purifying and cloning short oligonucleotides containing three perfect miR-27a binding sites into the *Sac*I and *Xba*I sites of the pmirGLO vector (Figure 1b). Primers used for the cloning of *ATM* wild type and mutated were:

ATM Fwd-5′-ATCTAGGAGCTCAGGAGTGGAAGAAGGCACTG-3′
ATM Rev-5′-ATCTAGTCTAGAACGCTGTCCAAAGTTTTTCC-3′
ATMdel1 Fwd-5′-AGTGGAAGAAGGCACTCTCAGTGTTGGTGGAC-3′
ATMdel1 Rev-5′-GTCCACCAACACTGAGAGTGCCTTCTTCCACT-3′
ATMdel2 Fwd-5′-CAAGGACAAATGAGGAGTAGTTAGATGAAAATATTAATCATAGAATAGTTGTT-3′
ATMdel2 Rev-5′-AACAACTATTCTATGATTAATATTTTCATCTAACTACTCCTCATTTGTCCTTG-3′

### 3.3. Transient Transfection and Cell Irradiation

Twenty-four hours prior to transfection, cells were plated in 3.5*-*cm culture dishes at 40%–60% confluence. A549 cells were transfected with pre-miR™ miRNA Precursor hsa-miR-27a (PM10939, Ambion Austin, TX, USA) by using Lipofectamine™ 2000 (Invitrogen Life Technologies, Carlsbad, CA, USA) for luciferase assays, or Hiperfect Transfection Reagent (QIAGEN, Hilden, Germany) for miRNA over-expression, according to manufacturer’s protocol. Mock-transfected cells underwent the transfection process without addition of miRNA (*i.e*., cells were treated with transfection reagent only). The medium was replaced 4–6 h after transfection with new culture medium. Transfections were performed in triplicate for each experiment and repeated 3–4 times. Cells were tested for miR or gene over-expression 24 h later.

Cell irradiation with γ-rays was performed at 24 h after transfection at the Department of Oncological and Surgical Sciences of Padova University with a _137_Cs source. miR-27a-transfected and untransfected cells were irradiated with 2 Gy (dose rate of 2.8 Gy/min). After irradiation, the medium was replaced with a fresh culture medium.

### 3.4. Total RNA Isolation and qRT-PCR

Total RNA was isolated by using Trizol^®^ Reagent (Invitrogen, Life Technologies, Carlsbad, CA, USA), according to the manufacturer’s protocol. Total RNA quantification was performed using the ND-1000 spectrophotometer (Nanodrop, Wilmington, DE, USA); RNA integrity and the content of miRNAs were assessed by capillary electrophoresis using the Agilent Bioanalyzer 2100, with the RNA 6000 Nano and the small RNA Nano chips, respectively (Agilent Technologies, Palo Alto, CA, USA).

The endogenous levels of mature miR-27a in untreated and in transfected A549 cells were determined by qRT-PCR using TaqMan MicroRNA Assay kit (Applied Biosystems, Foster City, CA, USA), that incorporate a target-specific stem-loop reverse transcription primer to provide specificity for the mature miRNA target. In brief, each RT reaction (15 mL) contained 10 ng of total purified RNA, stem-loop RT primer, RT buffer, 0.25 mM each of dNTPs, 50 U MultiScribe^™^ reverse transcriptase and 3.8 U RNase inhibitor. The reactions were incubated in a Mastercycler EP gradient S (Eppendorf, Hamburg, Germany) in 0.2 mL PCR tubes for 30 min at 16 °C, 30 min at 42 °C, followed by 5 min at 85 °C, and then held at 4 °C. The resulting cDNA was quantitatively amplified in 40 cycles on an ABI 7500 Real-Time PCR System, using TaqMan Universal PCR Master Mix and TaqMan MicroRNA Assays for miR-27a, and for U48 small nuclear (RNU48) as endogenous control.

For mRNA detection of *ATM* 1 μg of total RNA was retrotranscribed with ImProm-II Reverse Transcription System (Promega, Madison, WI, USA). qRT-PCR was performed with the Go Taq qPCR Master Mix (Promega, Madison, WI, USA) and gene-specific primers for *ATM* and *GAPDH* as reference. The relative expression levels of ATM and miR-27a were calculated using the comparative delta Ct (threshold cycle number) method (2-ddCt) implemented in the 7500 Real Time PCR System software (Applied Biosystems^®^ 7500 Real-Time PCR System, Life Technologies, Carlsbad, CA, USA, 2007) [47]. qRT-PCR reactions were always performed in quadruplicates.

### 3.5. Luciferase Reporter Assays

A549 cells were plated in 24-well plates (14 × 10^5^ cells/well) and 24 h later co-transfected with 50 ng of the pmirGLO dual-luciferase constructs, containing the 3′UTR of *ATM* gene, and with pre-miR™ miRNA Precursor hsa-miR-27a (miR-27a mimic) or pre-miR™ miRNA Precursor Molecules- Negative Control (Control mimic) (Ambion, Austin, TX, USA), using Lipofectamine^™^ 2000 (Invitrogen Life Technologies, Carlsbad, CA, USA). Lysates were collected 24 h after transfection and Firefly and Renilla Luciferase activities were consecutively measured by using Dual-Luciferase Reporter Assay (Promega, Madison, WI, USA), according to manufacturer’s instructions. Relative luciferase activity was calculated by normalizing the ratio of Firefly/Renilla luciferase to that of negative control-transfected cells.

### 3.6. Clonogenic Survival Assay

A549 (2 × 10^4^–4 × 10^4^ cells/cm^2^) were seeded in 3.5-cm culture dishes and allowed to attach overnight, then were subjected to transfection with miR-27a mimic and 24 h later irradiated with γ-rays. After irradiation, cells were harvested by trypsinization and counted by trypan blue dye exclusion. 250 viable cells were plated in 6-cm culture dishes in complete medium for the colony-forming assay and grown for 12 days before being stained with crystal violet for colony counting. Cell survival was calculated as percentage of cloning efficiency (CE) of transfected and untransfected cells irradiated with 2 Gy over CE of their respective non-irradiated control cells (*i.e.*, miR-27a + 2 Gy *vs.* miR-27a; 2 Gy *vs.* CTR).

### 3.7. Cell Cycle Analysis

Cells (1 × 10^6^) were harvested, fixed in 70% cold ethanol and stored at 4 °C overnight. Before analysis, cells were washed in distilled water, centrifuged and resuspended in 1 mL PBS containing 50 μg/mL propidium iodide (PI, Sigma-Aldrich, St. Louis, MO, USA) and 100 μg/mL RNAse, for DNA staining. Samples were incubated for 1 h at 37 °C and then analyzed using a BD FACSCanto™ II flow cytometer (BD Biosciences, San Jose, CA, USA). Data from 25 × 10^3^ cells/sample were collected for acquisition and cell cycle distribution analysis using CellQuest (Version 3.0, BD Biosciences, San Jose, CA, USA, 2007) and ModFit LT 3.0 softwares (BD Biosciences, San Jose, CA, USA, 2007), respectively.

### 3.8. Immunofluorescence Staining

A549 cells were grown on glass coverslips for 24 h, transfected with pre-miR-27a and 24 h later, irradiated. Control cells were subjected to the same treatments except for irradiation. At 0.5 h, 2 h, and 6 h after irradiation, cells were fixed in 4% formaldehyde (Sigma-Aldrich, St. Louis, MO, USA), at 37 °C for 15 min and washed twice with PBS. The cells were permeabilized with 0.2% Triton X-100 in PBS at 37 °C for 10 min and non-specific binding sites masked with goat serum (10% in PBS) at room temperature for 1 h. Cells were incubated for 1 h at room temperature with primary antibody anti-γ-H2AX (Ser139) (Abcam or Millipore Chemicon, Upstate Clone JBW301, 1:100). After three washes in PBS, cells were incubated with secondary antibody Alexa Fluor 488 goat anti-mouse (Life Technologies, 1:350), washed and counterstained with DAPI 0.2 μg/mL.

### 3.9. Statistical Analysis

Data are presented as means ± standard deviation (S.D.) from two to three independent experiments. All statistical comparisons were carried out by Student’s *t*-test and differences with a *p*-value < 0.05 considered significant.

## 4. Conclusions

In the present work, we examined the role of miR-27a during the DNA-Damage Response (DDR) after irradiation with γ-rays in A549 cells. The enforced expression of miR-27a in A549 cells altered cell proliferation, by increasing cells in S- and G_2_-phase. Over-expression of miR-27a decreased the ionizing radiation (IR) toxicity, as evidenced by the higher clonogenic survival and the reduced formation of early γ-H2AX foci in transfected cells compared with the control ones. These findings could be in part related to the action of miR-27a on *ATM* transcript and in part to the pleiotropic effect of miR-27a on target genes involved in cell proliferation and survival. On the whole, our results indicate that miR-27a promotes proliferation of A549 cells also after irradiation thus decreasing the potency of IR-DDR and probably affecting genome integrity. We cannot exclude, however, that the high basal glucose uptake rates in A549 cells due to the stimulation of epidermal growth factor receptor (EGFR) induced by IR and the EGFR-mediated increase in sodium/glucose cotransport-generated glucose uptake can promote the survival after IR [48] as observed in our experiments.
